# Pharmacist Role in Hypertension Management in the Community Setting: Questionnaire Development, Validation, and Application

**DOI:** 10.2147/PPA.S394855

**Published:** 2023-02-08

**Authors:** Lama Soubra, Ghada Elba

**Affiliations:** 1 Department of Biological and Environmental Sciences, College of Arts and Sciences, Qatar University, Doha, Qatar; 2 Pharmacy Practice Department, Faculty of Pharmacy, Beirut Arab University, Beirut, Lebanon

**Keywords:** pharmacist role, hypertension, hypertension management, questionnaire validation, pharmacist role questionnaire, community setting

## Abstract

**Background:**

Hypertension is a leading cause of mortality and morbidity globally. Pharmacists can play a substantial role in decreasing the burden of the disease.

**Purpose:**

The primary aim of this study was to develop and validate a scale assessing the pharmacist role in hypertension management in the community pharmacy setting. The secondary aims were to assess the services/interventions in hypertension management that were performed in the real-life setting, as well as the patient satisfaction from these services/interventions.

**Methods:**

This cross-sectional study was conducted in Egypt. The data were collected using a survey composed of three sections: a general section, the pharmacist role questionnaire section, and the patient satisfaction from the provided interventions/services section. The pharmacist role questionnaire was developed based on the pharmaceutical care practice conceptual model and included 23 questions. The face validity, content validity, reliability testing using Cronbach alpha, and construct validity using exploratory factor analysis were determined. The percentage of the frequency by which each role was reported to be performed was determined. Patient satisfaction from the provided interventions/services was determined by means of an overall rating. The correlation between practiced roles and patient satisfaction with received interventions/services was determined.

**Results:**

The questionnaire was valid with a 4-factor structure and a Cronbach alpha >0.75, reiterating the main pharmaceutical care practice domains: medication management, disease-state education, disease-state management, and care plan monitoring. Roles falling in the domains of disease state management and disease state education were significantly more practiced than roles falling in the other domains.

**Conclusion:**

Pharmacist practice in hypertension management in the community setting was inclined towards contemporary roles, such as disease state education and management. Patients seem to be satisfied with these roles.

## Graphical Abstract

**Figure:**
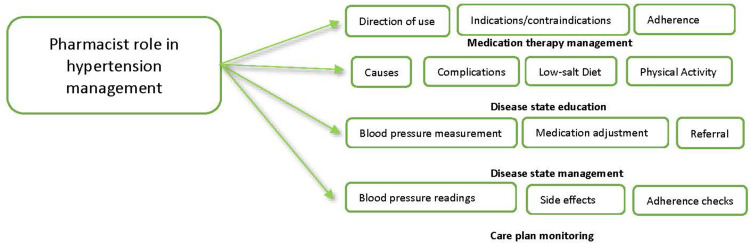

## Introduction

Cardiovascular diseases are the leading cause of mortality and morbidity worldwide.^1^ Hypertension, defined as elevated blood pressure (BP) (systolic BP ≥140 mmHg and/or diastolic BP ≥90 mmHg), is a major modifiable risk factor for cardiovascular diseases.^2^ Hypertension affects 1.28 billion adults aged 30–79 years worldwide, most (two-thirds) living in developing countries, posing thus a significant burden on the healthcare systems of these countries.^3^,^4^ The increasing burden of hypertension in developing countries was largely attributable to the aging of the population, behavioral factors, urbanization, unhealthy diets, obesity, social stress, and inactivity.^5^,^6^

In an attempt to reduce hypertension prevalence and improve its treatment and control, evidence-based clinical guidelines were developed. These guidelines focused on setting a holistic approach for hypertension management that encompasses screening strategies, blood pressure target goals, treatment modalities, and lifestyle modifications.^2^,^3^,^7^,^8^ Besides, a number of effective antihypertensive medicines and drug combinations are available in the markets. These include beta blockers, angiotensin converting enzyme inhibitors (ACEI), Angiotensin Receptor blockers (ARBs), calcium channel blockers (CCBs), nonsteroidal dihydropyridine-based mineralocorticoid receptor antagonists (MRAs) such as eplerenone and Finerenone and dual angiotensin II receptor-neprilysin inhibitors (valsartan with sacubitril).^9^

However, despite these guidelines and available therapies, only 1 in 5 hypertensive adults (21%) have their blood pressure adequately controlled, with the lowest control rates observed in developing countries.^3^,^4^,^10^,^11^ These low control rates were attributed to a suboptimal prescription of therapies, lack of disease state awareness, treatment inaccessibility, treatment non-adherence, and inadequate monitoring and follow-up.^12–18^

Uncontrolled hypertension can cause significant complications, such as but not limited to, congestive heart failure, myocardial infarction, angina, left ventricular hypertrophy, arrhythmias, stroke, and kidney failure.^2^,^3^,^7^,^8^ These complications would significantly affect the patient’s quality of life (QoL) and increase healthcare expenditure.^2^,^3^,^7^,^8^

Being the most accessible health care provider, the pharmacist can play a substantial role in the care of patients with chronic diseases and namely hypertension.^19–23^ Pharmacists can contribute to hypertension management in various ways, including disease state education, patient counseling, blood pressure measurement, and monitoring, adherence monitoring, and medication therapy management.^23–27^ Most of the studies evaluated the impacts of specific pharmacist interventions/services on hypertension control. The current practices of community pharmacists in hypertension management in the real-life setting remain poorly understood. Besides, there is a lack of standardized scales that would be used to assess the impacts of the community pharmacist interventions comprehensively.^28^ Furthermore, programs addressing hypertension management pitfalls are needed in developing countries to slow the rise of its absolute burden.^4^,^5^,^29^ Therefore, research to understand the role of pharmacist in hypertension management in the community setting is needed to develop and test effective, equitable, and sustainable interventions for implementing evidence-based clinical guidelines and public health policies worldwide.^4^ Hence, this study was conducted with a primary aim to develop and validate the pharmacist role in hypertension management questionnaire in the community setting. The secondary aims were to assess the pharmacist role in hypertension management in the real-life community setting, as well as the patient satisfaction from the provided interventions/services.

## Materials and Methods

### Study Design

This is a cross-sectional survey-based descriptive study that was conducted from November 2018 to June 2019, in Alexandria, Egypt.

### Data Collection Instruments and Procedures

A survey was developed to serve the purpose of this study. It consisted of three sections and was initially developed in the English language and then translated to the Arabic language. The first section of the survey, which is a general section, collected patient data and disease state-related information. It included 11 items varying between open and closed ended questions depending on the nature and the objective of the item. Collected data included age, gender, education level, number of medications taken daily, frequency of visiting the physician for follow-up on hypertension, and duration since diagnosis with hypertension. The second section tackled the pharmacist role in the management of hypertension in the community setting and was developed and validated initially before its administration to the final sample of participants. The last section aimed to assess the patient satisfaction from the pharmacist interventions/services. It included 6 items, rated from 1 to 5 (1 (strongly disagree to 5 (strongly agree)) based on the extent of agreement with the item statement included in this section. The statements were the followings: (1) It is easy to access my pharmacist when I have a question related to my hypertension condition or medications, (2) my pharmacist gives enough time to explain my hypertension condition and medications, (3) my pharmacist provides me with information on my hypertension condition and medications in a way that I can understand, (4) my pharmacist provides me with appropriate support to solve my hypertension-related issues, (5) my pharmacist serves my best interest, (6) I am overall satisfied with the interventions/services provided by my pharmacist.

The pharmaceutical care practice model was used as the conceptual framework for the development of the pharmacist role questionnaire. Based on this conceptual framework, four main role domains were identified for the pharmacist. These included medication management, disease state education, disease state management, and care plan monitoring. Preliminary relevant items for each of the four domains were drafted based on the United States pharmacopeia (USP) medication counselling behavior guidelines,^30^ similarly published questionnaires on pharmacists’ role,^31–35^ and the essential counselling points, questions and communication skills that the pharmacist should adopt with hypertensive patients.^26^ This yielded a total of 23 items that were included initially in the questionnaire that addressed the pharmacist role in the four practice domains, ie medication management, disease state education, disease state management, and care plan monitoring. The possible responses for each item were set following a 5-point Likert-scale model based on the frequency on which the intervention/service specified in the item was provided by the community pharmacists to their hypertensive patients.

The survey was administered in simple Fusha Arabic since it was used in an Arabic speaking community. All questions of the role of the pharmacist questionnaire section were developed initially in English and then translated from English to Arabic and then back translated to English. The forward and backward translation were conducted on two separate stages. First, members of the research group performed the process of forward and backward translation and then the original survey was sent to an independent translation agency for another trial of forward and backward translation. The two versions of the final translated survey were compared, and discrepancies were addressed before its use.

### Participants and Recruitment

Adult individuals aged 18 years old or more, visiting community pharmacies, outpatient hospital clinics, private clinics and public places including social clubs and city malls all over Alexandria were approached randomly by a member of the research team. They were asked whether they have hypertension and were treated with at least one antihypertensive medication for a duration of 1 month or more. If they were so, they were explained the purpose of the study and invited to participate. The recruitment was a multistage procedure. Firstly, lists of pharmacies and clinics were obtained from the pharmacy syndicate. List of malls was obtained from the municipality office. Pharmacies, outpatient hospital clinics, private clinics, social clubs, and city malls were selected from each geographical area by random sampling. Each centre was visited several days in a row during two time periods (morning or evening shifts) for 4–6 hours/ period, until recruiting the target number of patients/center. Selection of the appropriate sample size for validation is controversial, since the ratio of participants to item would range from 5:1 to 30–1 and samples range from 100 to 1000 participants.^36^ Our sample size was calculated using the 5–10 participants per item ratio with a minimum of 200 participants for questionnaire validation based on previous studies suggestions.^36^ The questionnaires were self-administered to avoid emotional pressure and untruthful answers when the participants were interviewed by the researchers. For illiterate participants, the questions were read as they are written clearly and out loud to the participants.

### Psychometrics

#### Face Validity

The pharmacist role questionnaire was distributed among sixty experienced practicing community pharmacists and they were asked if the pharmacist role questionnaire truly assess their role in managing hypertensive patients. Twenty-one experienced pharmacists responded and agreed that all items truly assess their roles and had no major amendment on the questionnaire.

#### Content Validity

For this type of validity, 30 experts in the field of pharmacy practice education and practice were approached and only 10 agreed to participate. The questionnaire was e-mailed to the experts who agreed to participate in this study (academicians (5) and practicing pharmacists (5, different from those included in the face validity). They were contacted by the researchers and asked if the questionnaire covers all aspects of the pharmacist’s role in managing hypertensive patients and whether the included items truly assess the research concept. In addition, their feedback on the coherence of the questionnaire, the order of the questions, the relevance, difficulty, and clarity of the items was collected. Only minor modifications were suggested at this stage. Besides, it was recommended to putting emphasis that the questionnaire is addressing the pharmacist’s and not the pharmacy technician’s role. This was taken into consideration while administering the questionnaire to the patients by verbally emphasizing this to the participants.

#### Pilot Testing

The survey was pilot tested on a convenience sample of 30 antihypertensive patients who were not included in the final study sample. In the pilot survey, a response box inviting comments regarding the content and structure of the questionnaire was added. Feedback indicated that one item lacked clarity and required rewording.

#### Reliability Testing

The reliability of the pharmacist role questionnaire and of the overall survey was evaluated using Cronbach alpha, which is a statistical tool to assess the internal consistency of surveys and questionnaires. A Cronbach alpha value of 0.7 or greater was considered an indicator of adequate internal consistency.^36^

#### Construct Validity by Factor Analysis

Exploratory factor analysis was used to assess the construct validity of the questionnaire and to calculate the factor loading of each component. The exploratory factor analysis determined the numbers of factors appropriate to our questions and categorized the questions according to their coherence to a specific factor. Factor analysis reduced the number of items by grouping the related items and identifying the unrelated items for removal. Principal axis factor method was used, and factors with Eigenvalue’s greater than one were chosen. To facilitate the interpretation of the factor structure, direct oblimin and Varimax rotation were both conducted with the latter being more reasonable since our variables are assumed to be uncorrelated and varimax yielded a simple structure which was easy to intercept. A Kaiser-Meyer Olkin measure and Bartlett’s test for sphericity were conducted to determine if the collected data were suitable for factor analysis. Before running a factor analysis, a correlations matrix of survey items was used to identify and remove highly correlated (>0.90) or weakly correlated (<0.30) items from the analysis. A cut-off value of 0.4 was used for the analysis of the communalities’ loadings.

### Assessment of the Pharmacist Role and Patient Satisfaction

After validation of the pharmacist role questionnaire section, the survey was further used to collect data aiming at assessing the pharmacist role in hypertension management, as well as the patient satisfaction from the provided interventions/services. Collected data were analyzed to derive the percentage of patients who reported to receive the intervention/service at a specific frequency for each questionnaire item. Besides, a score was attributed for each Likert point level as follows: 2 if the intervention/service is performed every time the patient visits the pharmacist, 1 for sometimes, 0 for not sure, −1 for rarely, and −2 for never. The score for each role domain was then calculated by summing up the scores of the items included in this domain. An overall score for the whole pharmacist role questionnaire was also calculated. Based on possible total scores, the overall score was attributed to one of the following 4 quartiles: high score quartile for scores ranging between 23 to 44, average score quartile for scores ranging between 1 and 22, low score quartile for scores ranging between 0 and −22, and very low score quartile for scores ranging between −22 and −44. The percentage of questionnaires having scores falling into each quartile was then determined. Moreover, the overall rating for the patient satisfaction from the received interventions/services section was calculated. This calculation was done by summing up the rating of each item of this section. Finally, the correlation between the overall scores of the pharmacist role questionnaire and the overall patient satisfaction from the pharmacist interventions/services rating was evaluated.

### Ethical Consideration

The study was reviewed and approved by the Beirut Arab University institutional review board (IRB) with code 2017H-001-P-R-0003 and complies with the declaration of Helsinki. Approval from the participating institutions was also obtained. Furthermore, all participants had to provide a written consent prior to participating in the survey. Moreover, it was clearly stated to hypertensive patients that the participation in the survey is voluntary, that they can withdraw from the study at any point in time, and that collected data will be treated anonymously, with confidentiality, and used to only serve the study purpose.

### Data Analysis

IBM SPSS Statistics software version 27 was used for all the statistical analysis. Besides the exploratory factor analysis, data derived from the survey were analyzed using descriptive statistics. Percentages were derived for categorical data and were compared using Kruskal Wallis test. Means with standard deviations were determined for continuous variables (overall rating scores and scores of the pharmacist role in each domain) and compared using the Student’s *t*-test (when comparing two groups) and ANOVA (when comparing more than two groups) with post-hoc analysis. Pearson correlation and regression analysis between the overall scores of the pharmacist role questionnaire and the rating scores of the patient satisfaction from provided interventions/services section was done.

## Results

### Patient Information

A total of 36 centers were included in this study, distributed as follows: 2 social clubs, 2 city malls, 8 clinics and 24 pharmacies. A total of 650 hypertensive patients were identified, out of which 460 patients accepted to participate in this study. Forty-eight filled surveys were excluded from further analysis because of missed or incomplete data. The first two hundred and fifty surveys were used to validate the role of the pharmacist section of the survey. Patients’ characteristics and disease state information collected from the first section of the survey are presented in Table 1. The mean age (SD) of the participants was 54 (±12.95) years old and 51% (237) were male. Most of the patients completed their bachelor’s degree and had a job at the time of the survey. The majority of the patients (93%) reported to have an easy access to retail pharmacies. Around 52% (211) of the patients were diagnosed with hypertension 5 years or less with 67% (275) being controlled with only one antihypertensive drug. Around 59% (242) were taking their antihypertensive medications once per day and 36.52 (151) twice per day. Around 74% (264) of the patients visited their physicians yearly or twice a year to follow-up on their condition. The most common co-morbid conditions were dyslipidemia followed by diabetes, in 17% (71) and 15.41% (63%), respectively. Around 46% (210) of patients had a BMI within the obese range (ie, above 30).

**Table 1:** Characteristics and Disease State Information Among Patients with Hypertension in Alexandria, Egypt from November 2018 to June 2019 (N = 460)

Age Mean ± SD (Years)	54.36±12.95
**Gender n (%)**	
Male	237 (51.5)
Female	223 (48.5)
**Education level n (%)**	
PhD	6(1.4)
MSc/diploma	17(4)
Middle	36 (8.6)
BSc	280 (68)
High school	51 (12.2)
Primary	3(0.8)
Illiterate	21(5)
**Body Mass Index (BMI) n (%)**	
<18.5 (Underweight)	0 (0)
18.5–24.9 (Healthy Weight)	60 (13)
25.0–29.9 (Overweight)	190 (41.3)
30.0 and Above (Obese)	210 (45.6)
**Easiness of accessibility to a pharmacy n (%)**	
Yes	384 (93.2)
No	28 (6.8)
**Duration of hypertension (in years) n (%)**	
<5	211 (51.2)
5–10	83(20.2)
10–15	37 (9.1)
15–20	48 (11.5)
>20	33 (8.0)
**Number of Hypertension medications (%)**	
1	275 (66.8)
2	127 (30.8)
3	10 (2.4)
**Frequency of taking medication n (%)**	
Once per day	242 (58.7)
Twice per day	151 (36.5)
3 times per day	20 (4.8)
**Use of a drug organizer n (%)**	
Yes	225 (54.6)
No	187 (45.4)
**Frequency of visiting the doctor to follow-up on hypertension n (%)**	
Monthly	15 (3.73)
Every two months	1 (0.3)
Twice per year	193 (46.76)
Yearly	71 (17.27)
When needed	1 (0.3)
Rarely	131 (31.85)
**Family history of hypertension complications n (%)**	
Yes	178 (43.19)
No	234 (56.80)
**Comorbidities (%)**	
None	161 (39)
**One comorbidity**	
Dyslipidemia	71 (17.1)
Diabetes	63 (15.4)
Heart failure	24 (5.8)
Others*	11 (2.7)
**Two comorbidities**	
Heart failure and dyslipidemia	23 (5.5)
Diabetes and dyslipidemia	44 (10.7)
**Three comorbidities**	
Dyslipidemia, heart failure and diabetes	16 (3.8)

**Note**: *Thyroid diseases, asthma, COPD.

### Validation of the Questionnaire

The total number of items of the questionnaire was 23 yielding as 11:1 ratio of variables to participants. The Kaiser-Meyer-Olkin (KMO) was 0.913 which is high indicating adequate sample size and the Bartlett’s test of sphericity was <0.001 which is statistically significant. Twenty-two out of 23 items were above 0.4, which is the cut off used in the analysis. An eigenvalue greater than 1 yielded a 4-factor structure with a cumulative variance of 54.8%. These four factors were the four domains of the pharmacist roles ie, medication management, disease-state education, disease-state management and care plan monitoring. Factor loading of items into their respective domains after varimax rotation is presented in Table 2. Finally, the Cronbach’s alpha coefficient value for the survey subsections and for the overall survey were above 0.75 demonstrating that the survey is reliable.

**Table 2:** Exploratory Factor Analysis Showing the Four Domains of the Community Pharmacist Roles Together with Their Factor Loading into Their Respective Domain

Domains	Description of Items	Item Loading
**Domain one: Medication management**	My pharmacist gives me clear directions on how to take my High Blood Pressure medication(s)	0.524
	My pharmacist explains what to expect from my High Blood Pressure medication(s) in terms of effect (How my Blood Pressure medications work) and side effects	0.539
	My pharmacist explains to me what to do in case of a missed dose of my High Blood Pressure medication(s)	0.606
	My pharmacist makes sure that I understand how to take my High Blood Pressure medication(s) and may ask me to repeat them	0.519
	My pharmacist helps me in managing my High Blood Pressure medication problems like side effects/ missed doses/cost/medication shortage/insurance coverage	0.649
	My pharmacist calls my healthcare provider to change my high Blood Pressure medication(s) in case of cost/coverage/shortage/side effects issues	0.730
	My pharmacist checks on my adherence to my High Blood Pressure medication(s)	0.521
	My pharmacist provides me with ways/tools (pill organizers, pill cards, reminder charts, mobile app,) to help me adhere to my HBP medications	0.536
**Domain Two: Disease-state education**	I receive education on High Blood Pressure causes and symptoms from my pharmacist	0.629
	My pharmacist explains to me High Blood Pressure complications clearly	0.650
	My pharmacist explains to me the roles of lifestyle modifications and medications in high blood pressure management	0.543
	My pharmacist explains to me the importance of physical activity on High Blood Pressure management	0.668
	My pharmacist explains to me the importance of following a low salt and low-fat diet on High Blood Pressure management	0.649
	My pharmacist provides me with materials (leaflets, brochures) on my High Blood Pressure condition to help understanding my condition	0.685
**Domain Three: Disease-state management**	My pharmacist measures my blood pressure	0.810
	My pharmacist tells me my target blood pressure values	0.541
	My pharmacist emphasizes on the importance to adhere to my BP medication(s) and life style modifications even if my Blood Pressure readings were within target values	0.531
	My pharmacist refers me to my health care provider when my Blood Pressure readings are outside the target limits	0.581
	My pharmacist helps me find solutions for my High Blood Pressure related concerns/issues	0.409
**Domain Four: Care plan Monitoring**	My pharmacist reminds me to measure my Blood Pressure at home and repeats important points on how and when to measure it	0.768
	My pharmacist provides me with materials/tools to record my Blood Pressure readings to monitor the effectiveness of my High Blood Pressure medication(s)	0.675
	My pharmacist provides me with materials/tools to monitor adherence to my High Blood Pressure medication(s) and their side effects	0.520

### Pharmacist Roles in Hypertension Management

#### First Domain: Medication Management

Table 3 presents the role of the pharmacist in the first practice domain, which is medication management. The roles that were reported to be significantly more practiced (ie at each encounter or sometimes) by pharmacists than not (ie when the patient asks or rarely/never), were making sure their patients understood the directions on how to take their HBP medications in 70% of responses versus 21% (p-value <0.01). This was followed by giving them clear directions on how to take their HBP medications and checking their adherence to their HBP regimens in 52% of responses versus 42%, and 51% responses versus 43% respectively; p-value <0.01. On the other hand, it was found that the pharmacist is significantly less likely to call the healthcare provider to change their patients’ high Blood pressure medication(s) in case of cost/coverage/shortage/side effect issues (28.72% versus 65%; p-value<0.01).

**Table 3:** Percentage Distribution of Responses for the Roles of the Community Pharmacist in Medication Management Among Patients with Hypertension in Alexandria, Egypt from November 2018 to June 2019 (N = 460)

	At Each Encounter (2)	Sometimes (1)	Cannot Remember (0)	Only When I Ask (−1)	Rarely/Never (−2)	P- value*
**My pharmacist gives me clear directions on how to take my High Blood Pressure medication(s)**	**26.4**	**25.2**	**5.7**	**25.7**	**16.9**	<0.01**
**My pharmacist explains what to expect from my High Blood Pressure medication(s) in terms of effect (How my Blood Pressure medications work) and side effects**	11.5	31.0	5.8	25.1	26.7	<0.01
**My pharmacist explains to me what to do in case of a missed dose of my High Blood Pressure medication(s)**	11.7	24.4	10.0	35.3	18.6	<0.01
**My pharmacist makes sure that I understand how to take my High Blood Pressure medication(s) and may ask me to repeat them**	**44.6**	**25.4**	**9.0**	**11.0**	**10.0**	<0.01**
**My pharmacist helps me in managing my High Blood Pressure medication problems like side effects/ missed doses/cost/medication shortage/insurance coverage**	18.3	18.3	8.8	28.5	26.1	<0.01
**My pharmacist calls my healthcare provider to change my high Blood Pressure medication(s) in case of cost/coverage/shortage/side effects issues**	8.1	21.1	5.3	11.9	53.6	<0.01
**My pharmacist checks on my adherence to my High Blood Pressure medication(s)**	**23.7**	**27.8**	**5.1**	**6.1**	**37.3**	<0.01**
**My pharmacist provides me with ways/tools (pill organizers, pill cards, reminder charts, mobile app,) to help me adhere to my High Blood Pressure medications**	23.4	25.1	3.7	18.3	29.5	0.1

**Notes**: *P-value obtained from Kruskal Wallis test. **Bolded values represent the more frequently practiced roles than not for each item in the practice domain.

#### Second Domain: Disease State Education

Table 4 presents the roles of the pharmacist in the second practice domain, which is the disease state education. The roles that were reported to be significantly more practiced by pharmacists (ie at each encounter or sometimes) by pharmacists than not (ie when the patient asks or rarely/never), were educating their patients about their disease state causes and symptoms in more than 62% of the responses versus 36%; p-value <0.01, as well as discussing the life style modifications and diet that they should follow (54% versus 38%; p-value <0.01). Whereas, pharmacists were reported to significantly less likely give their patients flyers and brochures to understand their condition (18% versus 75%; p-value <0.01) and to explain the importance of physical activity on HBP, and the complications of HBP (34% versus 57% and 38% versus 54%; p-value <0.01).

**Table 4:** Percentage Distribution of Responses for the Roles of the Community Pharmacist in Disease State Education Among Patients with Hypertension in Alexandria, Egypt from November 2018 to June 2019 (N = 460)

	At Each Encounter (2)	Sometimes (1)	Cannot Remember (0)	Only When I Ask (−1)	Rarely/Never (−2)	P-value*
**I receive education on High Blood Pressure causes and symptoms from my pharmacist**	**19.7**	**41.8**	**2.7**	**18.7**	**17.0**	<0.01**
**My pharmacist explains to me High Blood Pressure complications clearly**	9.9	28.6	7.8	30.3	23.5	<0.01
**My pharmacist explains to me the roles of lifestyle modifications and medications on high Blood Pressure management**	**21.0**	**28.5**	**6.4**	**15.3**	**28.9**	<0.01**
**My pharmacist explains to me the importance of physical activity on High Blood Pressure management**	12.9	21.1	7.0	13.8	45.2	<0.01
**My pharmacist explains to me the importance of following a low salt and low-fat diet on High Blood Pressure management**	**24.8**	**28.9**	**8.5**	**12.9**	**24.8**	<0.01**
**My pharmacist provides me with materials (leaflets, brochures) on my High Blood Pressure condition to help understanding my condition**	5.1	12.2	9.2	19.1	54.4	<0.01

**Notes**: *P-value obtained from Kruskal Wallis test. **Bolded values represent the more frequently practiced roles than not for each item in the practice domain.

#### Third Domain: Disease State Management

Table 5 presents the roles of the pharmacist in the third practice domain, which is the disease state management. In this domain, all roles were reported to be significantly more practiced by pharmacists (ie at each encounter or sometimes) by pharmacists than not (ie when the patient asks or rarely/never), with the exception of helping patients to find solutions to their high blood pressure-related concerns and issues, which was s reported among the study population (47% receiving the service versus 47% not receiving the service).

**Table 5:** Percentage Distribution of Responses for the Roles of the Community Pharmacist in Disease State Management Among Patients with Hypertension in Alexandria, Egypt from November 2018 to June 2019 (N = 460)

	At Each Encounter (2)	Sometimes (1)	Cannot Remember (0)	Only When I Ask (−1)	Rarely/Never (−2)	P-value*
**My pharmacist measures my Blood Pressure**	**39.6**	**18.0**	**4.1**	**31.6**	**6.8**	<0.01**
**My pharmacist helps me find solutions for my High Blood Pressure related concerns/issues**	19.0	28.6	5.1	23.7	23.6	0.1
**My pharmacist tells me my target Blood Pressure**	**28.2**	**29.9**	**6.5**	**18.7**	**16.7**	<0.01**
**My pharmacist emphasizes on the importance to adhere to my BP medication(s) and life style modifications even if my Blood Pressure readings were within target values**	**37.3**	**27.8**	**8.5**	**10.5**	**15.9**	<0.01**
**My pharmacist refers me to my health care provider when my Blood Pressure readings are outside the target limits**	**25.6**	**34.9**	**7.8**	**12.0**	**19.7**	<0.01**

**Notes**: *P-value obtained from Kruskal Wallis test. **Bolded values represent the more frequently practiced roles than not for each item in the practice domain.

#### Fourth Domain: Care Plan Monitoring

Table 6 presents the roles of the pharmacist in the fourth practice domain, which is the care plan monitoring. The pharmacists were significantly less likely to practice roles falling in this domain: providing materials for home monitoring of drug side effects and adherence (25% versus 68%; p-value <0.01), checking drug effectiveness (20% versus 70%; p-value <0.01), or reminding them to measure their blood pressure at home regularly (24% versus 70%; p-value <0.01).

**Table 6:** Percentage Distribution of Responses for the Roles of the Community Pharmacist in Care Plan Monitoring Among Patients with Hypertension in Alexandria, Egypt from November 2018 to June 2019 (N = 460)

	At Each Encounter (2)	Sometimes (1)	Cannot Remember (0)	Only When I Ask (−1)	Rarely/Never (−2)	P-value*
**My pharmacist reminds me to measure my Blood Pressure at home and repeats important points on how and when to measure it**	13.2	10.9	4.4	25.2	46.3	<0.01
**My pharmacist provides me with materials/tools to record my Blood Pressure readings to monitor the effectiveness of my High Blood Pressure medication(s)**	8.1	12.2	9.2	16.6	53.9	<0.01
**My pharmacist provides me with materials/tools to monitor adherence to my High Blood Pressure medication(s) and their side effects**	10.5	14.6	8.1	23.7	43.1	<0.01

**Note**: *P-value obtained from Kruskal Wallis test.

### Correlation Between the Pharmacist Role Questionnaire Overall Scores and the Patient Satisfaction Overall Ratings

The overall scores for the role of pharmacist questionnaire ranged between −44 and 44. As shown in Table 7, around 42% of the overall scores for the pharmacist role questionnaire were falling into the average score quartile, whereas around 50% of these were falling into the low and very low score quartiles. When considering each practice domain, pharmacists were practicing more roles falling into the disease-state management (62.58%), followed by disease-state education (41.83%), medication management (38.43%), and care plan monitoring (22.45%) (Table 8). Table 9 presents the mean (SD) rating and percentage of patients strongly agreeing/agreeing with the statements of the patient satisfaction survey. More than 65% were strongly agreeing/agreeing with the statements of the survey. There was no statistical difference between the ratings of the different items. Moreover, the mean (SD) of the overall rating for the patient satisfaction from the interventions/services provided by their pharmacist was 23.96 (4.25)/30.

**Table 7:** Percentage Distribution of the Overall Scores for the Community Pharmacist Role Questionnaire Among Patients with Hypertension in Alexandria, Egypt from November 2018 to June 2019 (N = 460)

Total Score Range	Percentage	P-value*
23 to 44	7.5	<0.01
1 to 22	41.8	
−22 to 0	29.6	
−23 to −44	21.1	

**Note**: *P- value obtained by One way ANOVA analysis.

**Table 8:** Percentage Distribution of the Scores for the Community Pharmacist Role Questionnaire Domains Among Patients with Hypertension in Alexandria, Egypt from November 2018 to June 2019 (N = 460)

**Domain 1** “Medication management”	**Score Range**	**Percentage**
	9 to 16	10.4
	1 to 8	27.8
	−9 to 0	32
	−8 to −16	29.8
**Domain 2** “Disease state education”	**Score Range**	**Percentage**
	7 to 12	8.8
	1 to 6	33
	−7 to 0	27.4
	−6 to −12	30.8
**Domain 3** “Disease state management”	**Score Range**	**Percentage**
	6 to 10	32.2
	0 to 5	30.2
	−6 to 0	21
	−5 to −10	16.6
**Domain 4** “Care plan monitoring”	**Score Range**	**Percentage**
	4 to 6	5.4
	1 to 3	17
	−4 to 0	22.1
	−6 to −3	55.5

**Table 9:** The Mean Rating (SD) and Percentage of Patients Strongly Agreeing/Agreeing with the Statements of the Patient Satisfaction Survey Among Patients with Hypertension in Alexandria, Egypt from November 2018 to June 2019 (N = 460)

Statement	Mean Rating (SD)/5	Percentage of Patients Agreeing and Strongly Agreeing
It is easy to access my pharmacist when I have a question related to their hypertension condition or medications	4.07(0.98)	77.8
My pharmacist gives enough time to explain my hypertension condition and medications	3.73(1.08)	66.3
My pharmacist provides me with information on my hypertension condition and medications in a way that I can understand	4.02±0.75	72.45
My pharmacist provides me with appropriate support to solve my hypertension related issues	4.14±0.75	80.82
My pharmacist serves my best interest	4.04±1.28	73.4
Overall satisfaction with the interventions/services provided by the pharmacist	3.95 (0.98)	71.78

**Figure 1:**
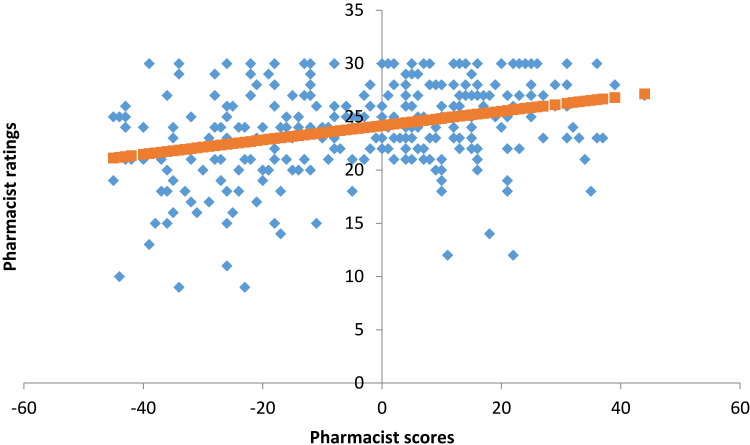
Correlation between the pharmacist role questionnaire overall scores and the patients satisfaction from the pharmacist services overall rating.

Figure 1 shows the correlation between the pharmacist role questionnaire overall scores and the patient satisfaction from the pharmacist services overall ratings. The linear regression shows a slope of 0.07 and an intercept of 24.2. Importantly, the regression analysis result indicates that there is a significant but weak relationship R^2^ = 0.103 (p < 0.001) between the overall scores of the pharmacist role questionnaire and the patient satisfaction from the pharmacist services overall ratings.

## Discussion

This study aimed to develop, validate, and apply a questionnaire that is able to assess the pharmacist role in the management of hypertensive patients in the community setting. The exploratory factor analysis demonstrated that the developed questionnaire has a four-factor structure that reiterated the pharmaceutical care practice model domains namely medication management, disease state education, disease state management, and care plan monitoring. The internal consistency test demonstrated that this questionnaire is a reliable tool for assessing the pharmacist role in hypertension management in the community setting.

Results of this study showed that the roles that pharmacists performed in hypertension management fall into the four practice domains of the pharmaceutical care model. However, the overall score assessment of the pharmacist role demonstrated that there are gaps in the performed pharmacist role in hypertension management, since more than half of the questionnaires had scores falling within the low and very low score quartiles. As identified by the score distribution for each domain, these role gaps were present in all domains, but were mainly related to the care plan monitoring and medication management domains. These gaps could generally compromise the pharmacist role in optimizing the patient’s care plan.^37^

Based on the results of this study, the community pharmacists practiced more roles that fell into the domains of disease state management and disease state education. This result suggests that there is a slight drift towards contemporary practice. This may be the result of the advancement of pharmacy education. Indeed, during the past decade, many pharmacy schools in Egypt have revamped their curriculum and introduced clinical pharmacy education courses.^38^ Besides, many clinical pharmacy degree programs were launched.^39^

Among the most practiced roles in the disease state management domain, pharmacists were measuring their patients’ blood pressure, telling them their target blood pressure levels, stressing on the importance to continue their therapies despite reaching their BP target levels, and referring them to their physicians when needed. These actions, namely measuring accurately, acting rapidly, and partnering with patients were deemed necessary to improve the patient hypertension control level.^40^ Besides, patients’ knowledge of their blood pressure target levels was found to be associated with improved blood pressure control.^41^ The pharmacists were also reported to engage in disease state education with their patients, emphasizing hypertension symptoms and the role of diet for the prevention and management of hypertension. Good patient disease knowledge was found to be significantly associated with controlled blood pressure, adherence to medication and healthy lifestyle behaviors, and improved outcomes in terms of hospital admissions.^42–44^ However, omitting explaining the hypertension complications might compromise the achievement of these outcomes, since a positive relationship between knowledge about hypertension complications and adherence was reported.^45^,^46^ Furthermore, another pitfall in that domain was not providing the patients with educational materials. Well-designed patient educational materials have greater impact on knowledge of the patients towards their disease management which would result in improvement of medication adherence.^47^,^48^ Besides, this omission would encourage patients to look for information by themselves and therefore, increasing the chances of searching non-evidence-based medicine resources.

As for the roles in the medication management domain, pharmacists tended to give their patients clear instructions on how to administer their medications, make sure that they understand these instructions, check their adherence to their medications, and provide them with tools that would help them to adhere to their medications. Simplifying the dosage regimen for patients was reported as a strategy to improve adherence.^49^,^50^ Moreover, in the absence of new antihypertensive drugs, addressing issues related to adherence to antihypertensive agents have been emphasized in recent guidelines as a means to optimize the use of current drug therapies.^49^,^50^ In addition, regular adherence checks by pharmacists was showed to improve systolic BP in patients with apparent treatment-resistant hypertension.^51^ Furthermore, using tools and a supportive, multidisciplinary team including pharmacists were found to help maintaining persistence with drug therapy. Although these roles, taken together, are essential to ensure that the patients would take their blood pressure medications as prescribed and, therefore, achieve good BP control levels, identified gaps in this domain would hinder the achievement of this outcome. Indeed, involving patients in decisions, discussing with them the pros and cons of medications, and helping them find solutions to their medication-related concerns are key factors for the success of drug therapy.^50^,^52^ Besides, overlooking these roles leaves patients ill-informed thus increasing the burden on the physicians. Patients who have medication-related problems not handled by their pharmacists visited their physicians instead, which would pose an unnecessary workload on the physicians.^53^ Finally, the role gap related to the communication with the patient healthcare provider indicated that pharmacists need to pay a closer attention to various consultative activities involving physicians. Effective communication and professional cooperation between pharmacists and physicians have been well known for reducing drug therapy-related problems and supporting optimized patient care.^54^,^55^

Despite that care plan monitoring is an essential component of the pharmaceutical care process, pharmacists seem to play very trivial roles in this domain. Care plan monitoring is useful for following the patient’s progress towards the achievement of goals of therapy and the desired outcomes and avoidance of potential adverse effects that would affect adherence to drug therapy.^56^ In addition, self-measured blood pressure when combined with clinical support (eg, one-on-one counseling, web-based or telephonic support tools, education), can enhance the quality and accessibility of care for people with high blood pressure and improve blood pressure control.^57^ Besides, medication adherence can significantly improve with a patient-centered approach, non-judgmental communication skills, and collaborative multidisciplinary management, including engagement of the patients in their care by self-blood pressure monitoring.^58^ Moreover, engaging patients in the monitoring process was found to increase self-esteem, feeling empowered, or independent, which would enhance service delivery and improve the patient outcomes.^59^ Therefore, taking an active role in this domain is deemed necessary to improve BP control and ensure medication adherence.^60^,^61^

Despite the above mentioned role gaps, patients seem to be satisfied from the interventions/services provided by their pharmacists related to the management of their hypertension, as evidenced by the overall rating of the patient satisfaction section. This result suggests that patients are appreciating the transition of the community pharmacist role from drug focus to pharmaceutical care practice. It supports the fact that the introduction of pharmaceutical care in pharmacies improves patient satisfaction.^62–64^ It also highlights the lack of awareness on what to expect and demand from the community pharmacist.^62^,^63^ Besides, despite the overall satisfaction from the pharmacist interventions/services, patients seemed to be unsatisfied by the duration of the interaction with their pharmacists. Time pressures and workloads were previously reported to affect the quality of interventions/services provided by the pharmacist.^65^,^66^ The perception of “lack of time”, reported as a barrier for service provision, must be taken into consideration through both better management and task redistribution for providing additional contemporary professional services.^65^,^67^

## Strengths and Limitations

This research has several strengths, such as being one of few studies addressing the pharmacist role in the management of chronic diseases in the community setting of a developing country, being multicenter, and having data generated by a validated questionnaire.

This study has also several limitations. First, the cross-sectional and quantitative nature of the research impeded the investigation of causalities as well as reasons behind the pharmacist role pitfalls. Second, the patients were not randomly selected; thus results from this study cannot be generalized. Third, the questionnaire was self-filled by the participants, therefore recall bias could have affected the collected data. Moreover, the blood pressure readings for the patients were not recorded at the time of the survey administration. Assessment of the impacts of the pharmacist role on the disease control was not, therefore, possible. Finally, some patients were recruited from pharmacies. This can be considered as a point of strength since recall bias would have been minimized in patients who had just received pharmacist services. This also can be considered as a point of weakness, since the presence of the participants in the pharmacy during the filling of the survey would have posed some psychological pressure pushing them to exemplify the interventions/services to gratify their pharmacists.

Despite these limitations, this study has yielded information about the practices and gaps in the pharmacist role in hypertension management in the community setting. It also shed the light on the need for interventional programs that would address these gaps to optimize patient care of patients with hypertension.

## Conclusion

In conclusion, this research provided insights about the situation in a developing country (Egypt) concerning the pharmacist role in hypertension management in the community setting. Results of this study demonstrated that community pharmacists started to practice contemporary roles in the management of hypertensive patients. However, these roles are still incomplete, which would impede the optimization of patient care and thus the achievement of desired outcomes from hypertension management. This result calls for interventional programs that would address role gaps. Besides, it highlights the need for further research that investigate the reasons behind the gaps in the community pharmacist role and the barriers to the effective implementation of this role in hypertension management in the community setting.
